# Clinical Imaging of Choroid Plexus in Health and in Brain Disorders: A Mini-Review

**DOI:** 10.3389/fnmol.2019.00034

**Published:** 2019-02-12

**Authors:** Violaine Hubert, Fabien Chauveau, Chloé Dumot, Elodie Ong, Lise-Prune Berner, Emmanuelle Canet-Soulas, Jean-François Ghersi-Egea, Marlène Wiart

**Affiliations:** ^1^Univ-Lyon, CarMeN Laboratory, Inserm U1060, INRA U1397, Université Claude Bernard Lyon 1, INSA Lyon, Charles Mérieux Medical School, Oullins, France; ^2^CNRS UMR5292, INSERM U1028, BIORAN Team, Lyon Neuroscience Research Center, Université Claude Bernard Lyon 1, Lyon, France; ^3^CNRS, Lyon, France; ^4^HCL, Lyon, France; ^5^CNRS UMR5292, INSERM U1028, Fluid Team and BIP Facility, Lyon Neuroscience Research Center, Université Claude Bernard Lyon 1, Lyon, France

**Keywords:** choroid plexus, imaging, central nervous system, blood-CSF barrier, inflammation, neurological disease

## Abstract

The choroid plexuses (ChPs) perform indispensable functions for the development, maintenance and functioning of the brain. Although they have gained considerable interest in the last years, their involvement in brain disorders is still largely unknown, notably because their deep location inside the brain hampers non-invasive investigations. Imaging tools have become instrumental to the diagnosis and pathophysiological study of neurological and neuropsychiatric diseases. This review summarizes the knowledge that has been gathered from the clinical imaging of ChPs in health and brain disorders not related to ChP pathologies. Results are discussed in the light of pre-clinical imaging studies. As seen in this review, to date, most clinical imaging studies of ChPs have used disease-free human subjects to demonstrate the value of different imaging biomarkers (ChP size, perfusion/permeability, glucose metabolism, inflammation), sometimes combined with the study of normal aging. Although very few studies have actually tested the value of ChP imaging biomarkers in patients with brain disorders, these pioneer studies identified ChP changes that are promising data for a better understanding and follow-up of diseases such as schizophrenia, epilepsy and Alzheimer’s disease. Imaging of immune cell trafficking at the ChPs has remained limited to pre-clinical studies so far but has the potential to be translated in patients for example using MRI coupled with the injection of iron oxide nanoparticles. Future investigations should aim at confirming and extending these findings and at developing translational molecular imaging tools for bridging the gap between basic molecular and cellular neuroscience and clinical research.

## Introduction

The choroid plexuses (ChPs) are small structures located in the lateral, third, and fourth brain ventricles. They are formed by numerous villi organized as a tight epithelium enclosing a highly vascularized stromal core that contains immune cells and fibroblasts. The ChPs produce the cerebrospinal fluid (CSF), form a protective barrier between the blood and the CSF (the blood-CSF barrier, BCSFB) and secrete various biologically active molecules. Hence, they play a vital role in maintaining the microenvironment in which the brain is located (Ghersi-Egea et al., 2018). The ChPs are also crucial for immune surveillance of the brain and provide a port of entry for immune cells in a range of neurological diseases (Schwartz and Baruch, 2014). The development of therapeutic strategies to protect the BCSFB may be helpful for the management of these diseases and is thus gaining a growing interest (Dragunow, 2013). However, to date, the role of ChP involvement in brain disorders is largely unknown, notably because their deep location inside the brain hampers non-invasive investigations. Imaging tools have become instrumental to the diagnosis and pathophysiological study of neurological and neuropsychiatric diseases. This review summarizes the knowledge gathered through clinical imaging of ChPs in health and neurological or neuropsychiatric disease not related to a primary ChP pathology.

## Methods

Supplementary Figure 1 presents the literature search flow chart. Supplementary Table 1 shows the summary of included studies. Note that the review does not include primary ChP pathology diseases such as ChP tumors, ventriculomegaly, and hydrocephalus. Results are discussed in the light of pre-clinical imaging studies whenever available.

## Results

### Morphology

Choroid plexuse size can be quantified with standard computed tomography (CT) and magnetic resonance imaging (MRI) approaches, and with ultrasound (US) in fetal or newborn infants. Contrast agent administration might be used to improve delineation. Madhukar et al. (2012) documented the size of normal ChPs in children between the ages of 0 and 16 years old. This study was intended to provide reference data to allow the detection of abnormal ChP size in developmental diseases, in the continuity of earlier studies that measured ChP size increase due to choroidal angiomatosis in children with Sturge-Weber syndromes (Stimac et al., 1986; Griffiths et al., 1996). However, it did not lead to further publications to date.

**FIGURE 1:**
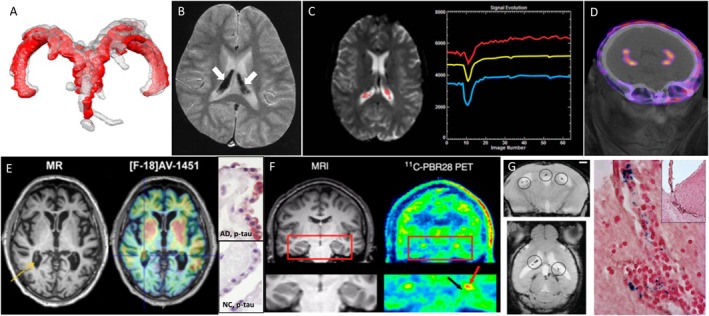
Examples of different clinical imaging techniques available for investigating the involvement of ChPs in brain disorders. **(A)** 3D reconstruction of ChP located within lateral ventricles space [red, before lumbar puncture (LP), and gray, after LP] in a patient with idiopathic intracranial hypertension (Lublinsky et al., 2018); **(B)** ChP iron deposition (arrows on hypointense signals) detected by susceptibility-weighted MRI in a patient who had received both ultrasmall superparamagnetic particles of iron oxide (USPIOs) 2 years earlier and multiple blood transfusions since (Daldrup-Link, 2017); **(C)** signal-time curves extracted in the ChP from dynamic susceptibility contras-enhanced MRI data: the yellow curve represents the mean signal calculated from all the pixels in the ChP volume, while the blue and red curves represent, respectively, the curve with lowest and highest baseline. After first passage of the gadolinium bolus (signal drop), the choroidal signals visually exceed the baseline which indicates gadolinium chelate extravasation into the ChP stroma (Bouzerar et al., 2013); **(D)** PET/CT images showing the normal ChP uptake of [^68^Ga]-DOTA-E-[c(RGDfK)]_2_ targeting α_v_β_3_ integrin (*This research was originally published in JNM.* Lopez-Rodriguez et al., 2016); **(E)** *in vivo* imaging using the Tau tracer [^18^F]AV-1451 showing high retention in AD patient and post-mortem ChP histopathology showing immunoreactivity in epithelial cells (pink color) with antibodies against pan-Tau in AD but not in normal control (NC) (Ikonomovic et al., 2016); **(F)** MRI and translocator protein (TSPO) labeling [^11^C]PBR28 PET images in a patient with left-sided temporal lobe epilepsy showing higher uptake in ipsilateral than in contralateral side in the ChP of lateral ventricles (red arrow) and hippocampus (black arrow) (*This research was originally published in JNM.* Hirvonen et al., 2012); **(G)** *in vivo* MRI using the very small particles of iron oxide (VSOP) and post-mortem Prussian Blue staining in a rat model of multiple sclerosis: VSOP (circles) was detected in the inflamed ChP at peak disease (Millward et al., 2013).

While performing an MRI morphometric brain analysis study in patients suffering from type 1 complex regional pain syndrome (CRPS), Zhou et al. (2015) serendipitously found a significant enlargement of ChPs compared with controls. The same phenomenon was observed with MRI in patients with idiopathic intracranial hypertension (IIH) (Figure 1A; Lublinsky et al., 2018). Using a different approach based on MRI texture analysis, Chaddad et al. (2017) identified ChPs as one of the regions having the most significant differences between patients with autism spectrum disorders (ASD) and controls. According to the authors, all these effects could result from the activation of several biological processes including the presence of edema and/or proliferation of ChP cells. Dedicated imaging techniques for evaluating CSF production (see below) may help investigate these hypotheses. Besides, if indeed increased ChP size observed by imaging techniques reflects an increase in ChP epithelial cell number, then it is likely that important choroidal functions other than CSF secretion, such as trophic factors and hormone carrier secretion, will be impacted as well.

Advanced MRI techniques such as diffusion-weighted/diffusion tensor imaging (DWI/DTI) can be used to detect microstructural tissue changes. DTI metrics have been measured in the ChPs of human subjects thus showing the feasibility of this imaging technique to characterize the ChPs despite their small size (Grech-Sollars et al., 2015). The apparent diffusion coefficient (ADC), reflecting the molecular motion of water in the interstitial space, was shown to increase with aging in ChPs (Alicioglu et al., 2017). According to the authors, this effect may be related to increased water diffusion across the epithelium via paracellular spaces, thus signaling BCSFB malfunction.

### Calcifications

The occurrence of macroscopic calcifications can be detected on CT and on T2^∗^-weighted MRI scans. ChP calcification increases in frequency and extent with age and is usually not associated with a pathology (Yalcin et al., 2016). However, Bersani et al. (1999) suggested a possible association between the size of ChP calcification and the severity of symptoms in schizophrenic patients independently of age, in line with a former study also based on CT-scan (Sandyk, 1993). The cause remains unclear and these results should be considered with caution as they have not been replicated to date.

### CSF Production

Because changes in CSF production rate likely contribute to pathological processes, as exemplified in Alzheimer’s disease (Silverberg et al., 2001; Serot et al., 2012), a validated imaging biomarker of CSF production may be valuable for investigating the involvement of ChPs in brain diseases. Net CSF flow through the cerebral aqueduct may serve as a marker of CSF production in the lateral ventricles, i.e., by the ChPs (Spijkerman et al., 2018). This flow may be estimated in humans using an phase-contrast MRI technique (Huang et al., 2004); however, the relationship between these measurements and CSF production has been recently questioned (Spijkerman et al., 2018). Of note, CSF production has choroidal and extrachoroidal components. The imaging of extrachoroidal CSF production and extraventricular CSF circulation is outside the scope of this review; however, because it is in close relation with CSF production at the ChPs, we briefly reviewed this literature in the Supplementary Data 1.

### Iron Deposits

Choroid plexuses are involved in iron exchanges between the blood and the brain (Morris et al., 1992; Deane et al., 2004; Rouault et al., 2009). They secrete transferrin (Leitner and Connor, 2012) and serve as an iron storage tissue (Lu et al., 1995; Rouault et al., 2009). MRI is a powerful tool to detect excessive iron in the brain. Several teams have reported iron deposition in ChPs of patients with transfusion-induced iron overload (Figure 1B; Kira et al., 2000; Qiu et al., 2014; Hasiloglu et al., 2017). Age-associated iron deposits were also documented in ChPs of mouse lemur, a non-human primate model of pathological brain aging (Joseph-Mathurin et al., 2013). The authors showed that immunization against Aβ worsened ChP iron deposits and suggested that MRI of human subjects immunized against Aβ should be evaluated to determine whether iron accumulation also occurred in humans.

### Capillary Perfusion and Permeability

The ChP blood flow is about fivefold higher than the cerebral blood flow (Maktabi et al., 1990). In contrast to the blood-brain barrier (BBB), the ChP capillaries are fenestrated and permeable, allowing free communication between the ChP stroma and the peripheral blood for molecules of different sizes. In physiological conditions, macromolecules with molecular weights up to ∼800 kDa or size ∼12 nm may diffuse into the ChP stroma (Strazielle and Ghersi-Egea, 2013). As a consequence, conventional contrast agents used in clinical MRI and CT (<2 kDa) diffuse into the stroma of ChPs, leading to homogenous enhancement (Guermazi et al., 2000). This physiological enhancement may be used to normalize abnormal brain enhancements (Miller et al., 2016; Vakil et al., 2017; Kim et al., 2018). In turn, Azuma et al. (2018) proposed a 4-point scale to score physiological enhancement of circumventricular organs including ChPs, on MRI, with the aim of recognizing abnormal enhancement in future investigations. Furthermore, ChP capillary permeability and perfusion may be quantified by dynamic contrast-enhanced/dynamic susceptibility contrast enhanced MRI (DCE/DSC-MRI) (Figure 1C; Artzi et al., 2013; Bouzerar et al., 2013; Vakil et al., 2017). This latter approach was used to show that ChP capillary permeability and perfusion decreased with aging (Bouzerar et al., 2013). In a mouse model of AD, stromal leakage of an iodinated liposome (size: 100–150 nm) was found in ChPs using microCT (Tanifum et al., 2014). However, the quantification of this leakage did not significantly differ compared to aged-matched control wild-type animals, thus suggesting that this phenomenon was independent from the pathology. Hence further studies are needed to determine whether the measurement of capillary perfusion and/or permeability at ChPs might serve as a biomarker in neurodegenerative diseases.

### BCSFB Permeability

The BCSFB at the ChPs is formed by the epithelium whose cells are sealed with tight junctions. As a consequence, no enhancement is seen in the CSF after contrast medium administration, unless the BCSFB is damaged. In patients, reports of contrast medium leakage into the brain ventricles are scarce (excluding cases of hemorrhage; Phatouros et al., 1999). A case of contrast leakage mimicking intraventricular hemorrhage was recently described in an ischemic stroke patient who had been treated with intravenous thrombolysis (IVT) (Park et al., 2017). A study investigating a small series of ischemic stroke patients undergoing mechanical thrombectomy in combination with IVT reported a blood-CSF arachnoid barrier disruption only (Renu et al., 2017). In pre-clinical ischemic stroke studies, two teams have reported MRI contrast medium leakage into the ventricles early after reperfusion (Nagahiro et al., 1994; Batra et al., 2010), in line with a previous study showing an increase in BCSFB permeability in rat models of focal cerebral ischemia (Ennis and Keep, 2006). Other brain pathologies that have been shown to display intraventricular conventional contrast medium extravasation in small animal models include Wernicke’s encephalopathy (Nixon et al., 2008), meningitis (Ichikawa et al., 2010), and experimental autoimmune encephalomyelitis (EAE), an animal model of multiple sclerosis (MS) (Wuerfel et al., 2010; Waiczies et al., 2012).

In turn, BCSFB integrity represents a hurdle for delivering drugs to the brain. The assessment of the drug-permeability barrier at the level of the ChP epithelium is therefore a desirable aim to understand cerebral pharmacokinetics. Non-metabolized radiotracers transported by both P-glycoprotein (ABCB1, also known as multidrug resistance protein) and multidrug resistant associated proteins (MRPs and ABCCs) have been used to this aim: the radiolabeled drug localized to ChPs with no detectable activity in adjacent CSF whether in healthy (Rao et al., 1999) or in epileptic subjects (Langer et al., 2007), such showing the efficacy of the BCSFB to prevent drug entry into the CSF. As MRPs rather than P-glycoprotein are expressed in ChPs both in rodent and humans (Gazzin et al., 2008), it is likely that the former plays a special role as biochemical barrier at ChPs. Another pre-clinical example is represented by the presence of ^64^Cu-labeled fusion protein etanercept (a TNF antagonist) into both the CSF and the ChPs following intravenous perispinal administration (Tobinick et al., 2009), thus demonstrating the potential of such techniques to investigate drug-concentration in both compartments.

### Receptor Imaging

In the literature, there are a number of PET studies that mention ChP non-specific uptake due to radiotracer extravasation or binding to calcification, which were thus excluded from this mini-review. A few studies, however, suggested specific ChP uptake of tracers targeting α_v_β_3_ integrins (Figure 1D; Minamimoto et al., 2015; Lopez-Rodriguez et al., 2016) or serotonin receptors (5-HTRs) (Ettrup et al., 2016; Schankin et al., 2016). An fMRI study also reported an increased BOLD signal in ChPs of volunteers administered with a 5-HT_2C_ receptor agonist (Anderson et al., 2002). This is in line with the known expression of these receptors in ChPs. Altogether these data suggest that imaging ChP receptors is feasible in humans.

### Proteinopathies

Proteinopathies can be explored with PET radiotracers targeted at aggregated proteins; yet, in these brain imaging studies, ChPs are usually disregarded because signal enhancement is thought to reflect non-specific uptake, also called “off-target binding” (Lowe et al., 2016). However, in the case of the recently developed Tau radiotracer flortaucipir (also known as [^18^F]AV-1451 or [^18^F]-T807), elevated ChP binding has attracted considerable interest because of ChP proximity (and potential signal contamination) to hippocampus, a key region for staging tauopathy in Alzheimer’s disease (AD) (Pontecorvo et al., 2017; Lee et al., 2018). At the moment, there are conflicting reports on the substrate of this radiotracer uptake in ChPs: while off-target binding to leptomeningeal melanocytes (adjacent to the lateral ventricles; Marquie et al., 2017), or to ChP calcifications (Lowe et al., 2016), have been suggested by autoradiographic studies, Ikonomovic et al. (2016) reported the histological detection of Tau protein aggregates in ChP epithelial cells, thus suggesting a possible “on-target” binding (Figure 1E). Therefore there is still an open debate regarding the interpretation of ChP signals in PET studies of proteinopathies.

In addition, the accumulation of aggregated proteins within epithelial cells may alter the ChP metabolic activity. Daouk et al. hypothesized that dynamic PET imaging with ^18^F-FDG could be useful to assess glucose metabolism as a marker of ChP epithelium activity in elderly adults, with a view to early diagnosis of AD. The FDG uptake in ChPs decreased with increasing disease severity, thereby providing the proof-of-concept that PET-FDG is feasible and useful to study ChP functional behavior in AD patients (Daouk et al., 2016).

### Immune Responses

The ChP stroma contains immune cells such as macrophages, neutrophils, dendritic cells, B and T cells and serves as a gateway for immune cell trafficking into the CSF (Ghersi-Egea et al., 2018). PET imaging of translocator protein 18 kDa (TSPO) is the method of choice for clinically evaluating neuroinflammation (Kreisl et al., 2016) as it is overexpressed in activated microglia/macrophages. In patients with unilateral temporal lobe epilepsy, a higher uptake of the TSPO radiotracer [^11^C]PBR28 was observed in the ChPs ipsilateral to the seizure focus (Figure 1F; Hirvonen et al., 2012). In a mouse model of chronic systemic inflammation, Drake et al. (2011) showed with histological experiments an intense recruitment of activated immune cells at ChPs, while in their parallel human study, they did not examine a potential uptake of the TSPO radiotracer ^11^C-PK11195 in ChPs of patients at risk of stroke, probably because of the expected “off-target” effect.

Alternatively, neuroinflammation may be assessed with MRI by labeling cells with a contrast agent. Reticuloendothelial MRI contrast media (such as superparamagnetic particles of iron oxide or SPIO) are taken up by phagocytic cells following their intravenous administration. SPIO-enhanced MRI has been extensively used to monitor phagocytic cells in subjects with neuroinflammatory diseases (Chauveau et al., 2011; Gkagkanasiou et al., 2016). With regard to ChPs, only pre-clinical data have been published to date. An accumulation of contrast material was observed in the ChPs of animal models of ischemic stroke (Wiart et al., 2007; Desestret et al., 2009; Henning et al., 2009) and EAE (Wuerfel et al., 2010; Millward et al., 2013, 2017). These results were obtained using contrast agents of various formulations and size (∼1000 kDa for Gadofluorine M and 7–150 nm for SPIOs). Contrast agents were found in the stroma (Figure 1G) with evidence of internalization by phagocytic cells (Wuerfel et al., 2010; Millward et al., 2017). Of note, the very small SPIOs (VSOP) were also found in the endothelium (Millward et al., 2013) and epithelium (Millward et al., 2017). Altogether, these data confirm ChPs involvement in neuroinflammatory diseases and suggest that SPIO-enhanced MRI might represent a powerful tool to study immune activation. Although clinical MRI studies have been conducted using ultrasmall (<50-nm) SPIO (USPIO) in patients with ischemic stroke (Cho et al., 2007; Nighoghossian et al., 2007) and MS (Tourdias et al., 2012), to the best of our knowledge, none has specifically examined the ChPs. Retrospective analysis of those data might help determine whether these pre-clinical observations have a translation in the clinical field.

## Summary and Future Directions

Despite an increasing interest, the ChPs are still relatively understudied using neuroimaging. One reason for this lack of data is the small size of ChPs, which may be even more problematic in neurodegenerative diseases due to atrophy (Serot et al., 2003). Another reason is the unspecific ChP uptake of clinically approved contrast agents in physiological conditions, thus complicating image interpretation in pathological conditions. In addition, partial volume effects and/or spill-over from adjacent tissues may biased the quantitative values measured in ChPs. For all these reasons, ChPs are still viewed as challenging structures to image.

**FIGURE 2:**
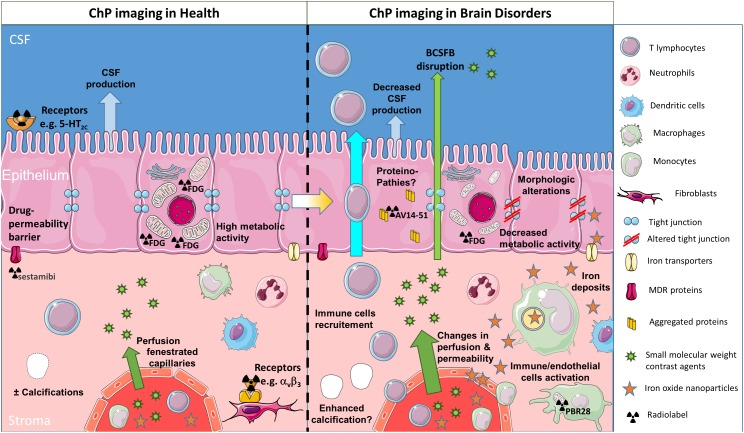
Schematic of different clinical imaging techniques available for investigating the involvement of ChPs in brain disorders. ChP volume and the presence of calcification may be assessed through conventional imaging techniques such as CT and MRI. CSF production may be evaluated with phase-sensitive MRI sequences. ChP capillary perfusion and permeability may be quantified with MRI using dynamic imaging coupled to small molecular weight contrast agents such as gadolinium chelates. Nuclear medicine techniques such as PET give access to: (i) the occurrence of receptors on stromal or epithelial cells (both at the basal or at the apical side) and (ii) epithelial cell metabolism, thanks to dedicated radiotracers. All of these morphological and functional features present a progressive degradation throughout the aging process which might get aggravated in neurological diseases. In addition, in pathological conditions, the extravasation of small molecular weight contrast agents into CSF may allow to image blood-CSF barrier disruption. In inflammatory conditions, reticuloendothelial MRI contrast agents such as iron oxide nanoparticles may get access to the ChP stroma and accumulate into endothelial, stromal, and/or epithelial cells. Alternatively, activation of stromal immune cells (whether resident or infiltrating) may be observed with TSPO-targeted PET radiotracers. Finally, the presence of aggregated proteins due to proteinopathies might be detected with dedicated PET tracers, although this is still debated due to “off-target” binding. ChPs, choroid plexuses; CSF, cerebrospinal fluid; BCSFB, blood-cerebrospinal fluid barrier; MDR, multidrug-resistant; CT, computed tomography; MRI, magnetic resonance imaging; PET, positron emission tomography; TSPO, translocator protein 18 kDa (this figure was prepared with Servier Medical Art).

Despite these limitations, there is a growing body of evidence showing that ChP imaging is feasible and valuable. Figure 2 presents imaging strategies that may be used to obtain information about ChPs morphology and function. To date, most clinical imaging studies of ChPs have used human subjects free from neurological disease to show the interest of different ChPs imaging biomarkers, sometimes combined with the study of normal neurodevelopment or aging. Very few have actually tested the value of ChP imaging biomarkers in patients with neurodiseases; but these pioneer studies gave promising results in CRPS, IIH, ASD, schizophrenia, epilepsy, and AD. Future investigation should aim at confirming and extending these findings, evaluating their clinical relevance, and using them to investigate the involvement of ChPs in brain disorders.

Retrospective analysis of neuroimaging databases with a focus on ChPs might provide some answers in this respect. Morphological and functional changes in ChPs may be readily estimated by MRI, CT, and PET imaging, all approaches being relatively widespread in clinical trials that make use of neuroimaging. The questions that need to be answered are related to the potential of the different imaging techniques to differentiate between normal and pathological conditions, to follow-up disease progression and to monitor the effects of therapy. In addition, the study of inflammation at the ChPs may bring some new insights into the role of these structures in neuro-inflammatory disorders.

There are obvious limitations to retrospective studies, especially for the study of ChPs, since by definition they were not optimized to image such small structures. In the future, prospective studies should thus be properly designed, by making use of the latest technological developments such as hybrid imaging with PET/MR and the development of dedicated imaging tools. In parallel, further pre-clinical investigation is needed to elucidate the biological correlates of ChPs imaging biomarkers.

## Conclusion

In summary, the clinical assessment of ChP alterations associated with brain disorders using neuroimaging methods may be the necessary step to effective therapeutic targeting of these conditions. The development of translational imaging approaches dedicated to the evaluation of ChP morphology and function could play a crucial role, by bridging the gap between basic molecular and cellular neuroscience and clinical research.

## Author Contributions

MW conceived the review focus, conducted the literature review, and overviewed drafting of the manuscript. VH, EC-S, FC, CD, EO, L-PB, and J-FG-E reviewed the literature in their respective fields of expertise, i.e., neuroscience, neuroimaging, neurology, neuroradiology, and plexus choroid research, and finalized the manuscript. All authors approved final version of manuscript.

## Conflict of Interest Statement

MW has a research contract with AMAG for the study of neuroinflammation using Ferumoxytol. The remaining authors declare that the research was conducted in the absence of any commercial or financial relationships that could be construed as a potential conflict of interest.

## Abbreviations

AD: Alzheimer’s disease
ASD: autism spectrum disorders
BCSFB: blood-CSF barrier
ChP: choroid plexus
CRPS: complex regional pain syndrome
CSF: cerebrospinal fluid
CT: computed tomography
IIH: idiopathic intracranial hypertension
MRI: magnetic resonance imaging
MS: multiple sclerosis
PET: positron emission tomography
US: ultrasound
