# Supplementary material for: Clinical Imaging of Choroid Plexus in Health and in Brain Disorders: A Mini-Review
Source: Front Mol Neurosci. 2019 Feb 12;12:34. doi: 10.3389/fnmol.2019.00034 (PMC6379459; doi:10.3389/fnmol.2019.00034)
Supplement: Supplementary file 1 [file Data_Sheet_1.pdf]

Supplementary Material

Clinical Imaging of Choroid Plexus Response in Health and in Brain Disorders: a Mini-Review

Violaine Hubert, Fabien Chauveau, Chloé Dumot, Elodie Ong, Lise-Prune Berner

Emmanuelle Canet-Soulas, Jean-François Ghersi-Egea, Marlene Wiart*

*** Correspondence:** Corresponding Author: marlene.wiart@univ-lyon1.fr

1 Supplementary Data: Imaging of Extrachoroidal CSF Production and perivascular CSF flow

Beside CSF production at the ChPs, it is now agreed that there are some extrachoroidal CSF sources. Water production from cell metabolism and water transfer across the blood-brain barrier contribute to the production of interstitial fluid, which in part drains into the CSF along white matter tracts and paravascular route. CSF entering the brain parenchyma to exchange with interstitial fluid before returning to the subarachnoid spaces also uses this paravascular route. The peri-arterioveinous CSF circulation is hypothesized to act as a clearance mechanism for interstitial fluid-borne metabolites such as β-amyloid, and is sometimes referred to as the glymphatic system (Abbott et al., 2018). In Alzheimer's disease (AD) patients, brain-to CSF [^{15}O]H_2O ratio has been quantified with PET imaging (Suzuki et al., 2015). Water influx into the CSF was significantly reduced in these patients, which, according to the authors, "may disturb the clearance rate of β-amyloid and therefore be linked to the pathogenesis of AD" (Suzuki et al., 2015). Water influx into and from CSF have been linked to the presence of water channels named aquaporins (AQPs) (Speake et al., 2003). On the one hand, the ChP epithelial cells are known to express AQP-1, which are thought to participate in CSF secretion (Kaur et al., 2016). On the other hand, AQP-4 is strongly expressed at the astrocytic end-feet

surrounding microvessels, at the glia limitans, and at the ependyma, and may play a role in CSF or edema resorption (Kaur et al., 2016). Astroglial AQP-4 expression appears somehow associated with the glymphatic system of fluid circulation, through a mechanism which is so far not well understood (Abbott et al., 2018). Imaging tools have been developed in an effort to better understand the role of AQP-4 in normal and diseased brain. The first report of AQP-4 PET imaging of the human brain has been recently published (Suzuki et al., 2013): the ChPs showed uptake of the radiotracer, a surprising result considering the low and controversial ChP expression of AQP-4 in normal brain. Otherwise the images generated matched the overall expression of AQP-4 in glial cell derived structures. This suggests that this imaging approach might be useful to design human studies allowing a better understanding of AQP-4 in brain diseases. Finally, the glymphatic system has been recently imaged by MRI after intrathecal injection of an MR contrast agent serving as a CSF marker in control individual and in patients with idiopathic normal pressure hydrocephalus-associated dementia (Ringstad et al., 2017; Eide and Ringstad, 2018; Ringstad et al., 2018). These clinical studies bring information on CSF-brain exchange in human and highlight changes in fluid dynamic in patients, thus opening the way for better understanding this unexplored system in neurological disorders.

References

Abbott N.J., Pizzo M.E., Preston J.E., Janigro D., Thorne R.G. (2018). The role of brain barriers in fluid movement in the CNS: is there a 'glymphatic' system? *Acta Neuropathol* Mar;135(3):387-407

Eide, P.K., and Ringstad, G. (2018). Delayed clearance of cerebrospinal fluid tracer from entorhinal cortex in idiopathic normal pressure hydrocephalus: A glymphatic magnetic resonance imaging study. *J Cereb Blood Flow Metab***,** 271678X18760974. doi: 10.1177/0271678X18760974.

Igarashi, H., Tsujita, M., Kwee, I.L., and Nakada, T. (2014). Water influx into cerebrospinal fluid is primarily controlled by aquaporin-4, not by aquaporin-1: 17O JJVCPE MRI study in knockout mice. *Neuroreport* 25(1)**,** 39-43. doi: 10.1097/WNR.0000000000000042.

Kaur, C., Rathnasamy, G., and Ling, E.A. (2016). The Choroid Plexus in Healthy and Diseased Brain. *J Neuropathol Exp Neurol* 75(3)**,** 198-213. doi: 10.1093/jnen/nlv030.

Ringstad, G., Valnes, L.M., Dale, A.M., Pripp, A.H., Vatnehol, S.S., Emblem, K.E., et al. (2018). Brain-wide glymphatic enhancement and clearance in humans assessed with MRI. *JCI Insight* 3(13). doi: 10.1172/jci.insight.121537.

Ringstad, G., Vatnehol, S.A.S., and Eide, P.K. (2017). Glymphatic MRI in idiopathic normal pressure hydrocephalus. *Brain* 140(10)**,** 2691-2705. doi: 10.1093/brain/awx191.

Speake, T., Freeman, L.J., and Brown, P.D. (2003). Expression of aquaporin 1 and aquaporin 4 water channels in rat choroid plexus. *Biochim Biophys Acta* 1609(1)**,** 80-86.

Suzuki, Y., Nakamura, Y., Yamada, K., Huber, V.J., Tsujita, M., and Nakada, T. (2013). Aquaporin-4 positron emission tomography imaging of the human brain: first report. *J Neuroimaging* 23(2)**,** 219-223. doi: 10.1111/j.1552-6569.2012.00704.x.

Suzuki, Y., Nakamura, Y., Yamada, K., Igarashi, H., Kasuga, K., Yokoyama, Y., et al. (2015). Reduced CSF Water Influx in Alzheimer's Disease Supporting the beta-Amyloid Clearance Hypothesis. *PLoS One* 10(5)**,** e0123708. doi: 10.1371/journal.pone.0123708.

2 **Supplementary Figure 1: Flow diagram of the selection process by which the studies were included in the mini-review article.** We searched the PubMed database for original articles written in English in the last 20 years (1998-2018) that dealt with the clinical imaging of plexus choroid in neuropathologies others than primary ChP diseases such as ChP tumors, ventriculomegaly and hydrocephalus. The majority of papers that were retrieved either dealt with primary ChP pathologies or mentioned the ChPs as a non-specific site of contrast agent uptake, hence the high number of rejected papers.

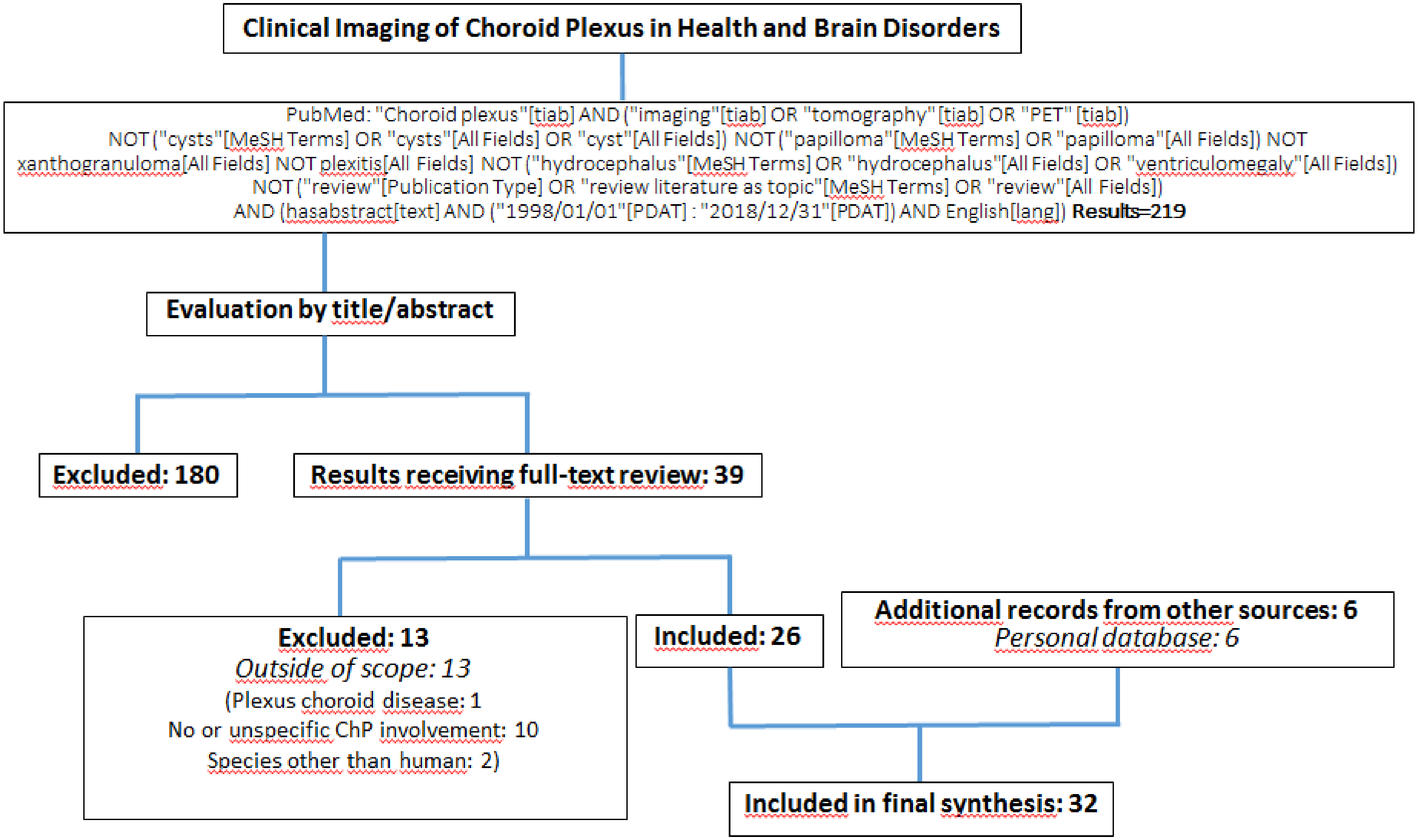

3 Supplementary Table 1: Summary of studies included in the mini-review article

Reference	Imaging technique	Subjects	Results
**Morphology**			
Madhukar et al (2012)	Contrast-enhanced MRI, US	90 healthy children (0-16 y.o.)	Report of ChP size in normal neurodevelopment; no impact of age or gender on size.
Zhou et al (2015)	3T MRI (T1w)	12 complex regional pain syndrome (CRPS) patients (W, 36-58 y.o)<br>12 healthy subjects (W, 25-60 y.o.)<br>8 patients with another chronic pain syndrome (2W, 8M, 38-72 y.o.)	Larger ChP volume in CRPS patients compared to control groups.
Lublinsky et al (2018)	1.5T MRI (MR venography and T1w)	18 idiopathic intracranial hypertension (IIH) patients (W, 27 ± 14 y.o.)<br>30 healthy subjects (27W, 3M, 26 ± 13 y.o.)	Larger ChP volume in IIH patients compared to the control group. Decrease of ChP volume after lumbar puncture
Chaddad et al (2017)	MRI (T1w)	539 autism spectrum disorder (ASD) patients (65W, 474M, 17± 8 y.o.)<br>573 controls (99W, 474M, 17± 8 y.o.)	Radiomic analysis of texture features showing differences in ChPs between ASD and control groups; no impact of age or gender on ChP texture features.
Grech-Sollars et al (2015)	1.5T or 3T MRI (DWI and DTI)	9 healthy subjects (2W, 7M, 25-34 y.o.) scanned on 8 scanners<br>202 patients without major cranial	The intra-scanner, inter-scanner and inter-volunteer coefficients of variation of DTI parameters (such as ADC and FA) were higher in ChPs than in other brain regions.

Alicioglu et al (2017)	1.5T MRI (DWI)	abnormalities (105W, 97M, 44± 24 y.o.)	Positive correlation between increased ADC values in ChPs and age
**Calcifications**			
Yalcin et al (2016)	CT	11,941 healthy patients (52% W, 47 ± 17 y.o. ; 48% M, 45 ± 18 y.o.)	ChP calcifications are frequent and increase with age
Bersani et al (1999)	CT	87 schizophrenic patients (M, 30 ± 7 y.o.)<br>46 healthy subjects (M, 29 ± 8 y.o.)	ChP calcification sizes are possibly associated with psychopathological features in schizophrenic patients regardless of age.
**CSF Production**			
Huang et al (2004)	1.5T Phase-Contrast-MRI	23 healthy subjects (5W, 18M, 21-39 y.o.)	Supratentorial average CSF production rate of 305 µL/min, in accordance with literature-reported values obtained with invasive measurements.
Spijkerman et al (2018)	7T Phase-Contrast-MRI	12 healthy subjects (5W, 7M, 19-39 y.o.)	Measurement of net CSF flow using PC-MRI is confounded by respiration effects.

**Iron Deposits**			
Kira et al (2000)	MRI (T2*w) (Field strenght not mentioned)	1 Diamond-Blackfan anemia patient (M, 7 y.o.)	Accumulation of iron in ChPs but not in brain parenchyma following blood transfusion.
Qiu et al (2014)	3T MRI (QSM)	31 transfusion-dependent β–thalassemia patients (17W, 14M, 25± 6 y.o.)<br>33 healthy subjects (16W, 17M, 26± 4 y.o.)	Higher susceptibility values reflecting an iron overloading in patient ChPs compared to control. No age effect on these values.
Hasiloglu et al (2017)	1.5T MRI (T2*w, T2w, SWI)	18 transfusion-dependent β–thalassemia patients (1W, 17M, 7-39 y.o.)<br>18 healthy subjects (ages not mentioned)	Among two others susceptibility-based approaches, SWI is the most reliable method for revealing iron deposition in ChPs of repeated transfused β–thalassemia patients.
**Perfusion & Permeability**			
Azuma et al (2018)	3T MRI (contrast-enhanced (CE) 3D T2-FLAIR)	30 patients with normal brain MRI (20W, 10M, 25-86 y.o.)	ChPs show the highest enhancement after the median eminence amongst circumventricular organs (using a 4-point grading system).
Artzi et al (2013)	3T DSC-MRI	20 healthy subjects (11W, 9M, 36 ± 13 y.o.)	Bolus arrival time is delayed in ChP vessels compared to other brain arteries and even veins.

Bouzerar et al (2013)	3T DSC-MRI	15 retrospective patients (7W, 8M, 21-68 y.o.)	ChP perfusion and permeability decrease with aging.
**BCSFB Permeability**			
Park et al (2017)	Contrast-enhanced CT, MRI (SWI)	1 patient with ischemic stroke (M, 66 y.o.)	Case report of contrast agent leakage in the lateral ventricles after intravenous thrombolysis.
Rao et al (1999)	[^{99m}Tc]-sestamibi SPECT	1 healthy subject (not documented)	No detectable sestamibi activity in CSF adjacent to ChPs despite an important retention in ChPs: the radiotracer does not cross the intact BCSFB.
Langer et al (2007)	[^{11}C]-verapamil PET	7 drug-resistant temporal lobe epilepsy patients (2W, 5M, 33-54 y.o.)	High [^{11}C]-verapamil retention in patient ChPs. No radiotracer activity in the adjacent CSF.
**Receptors imaging**			
Minamimoto et al (2015)	[^{18}F]-FPPRGD2 PET/CT	35 patients with different types of cancer (24W, 11M, 52 ± 11 y.o.)	ChP uptake of radiotracer targeted at integrin $\alpha_v\beta_3$.
Lopez-Rodriguez (2016)	[^{18}F] -FPPRGD2 PET/CT	5 healthy subjects (2W, 3M, 28-65 y.o.)	ChP uptake of radiotracer targeted at integrin $\alpha_v\beta_3$.
Ettrup et al (2016)	[^{11}C]-Cimbi-36 PET	16 healthy subjects (10W, 6M, 24 ± 6 y.o.)	ChP uptake of radioligand targeted at 5-HT_{2A} receptor.

Schankin et al (2016)	[11C]-DHE PET	6 migraineur patients (4W, 2M, 33 ± 7 y.o.) at rest and during acute glyceryl trinitrate-induced migraine attacks<br>6 healthy subjects (4W, 2M, 32 ± 9 y.o.)	No significant difference in ChP uptake of radiolabeled migraine medication DHE targeted at 5-HT receptors in migraineurs interictally or ictally and in control subjects
Anderson et al (2002)	BOLD pharmacoMRI	8 healthy subjects (M, 26-42 y.o.)	Administration of 5-HT_{2C} receptor agonist mCPP increases signal in ChPs.
**Metabolic activity**			
Daouk et al (2016)	dynamic [18F]-FDG PET	17 Alzheimer's disease (AD) patients (12W, 5M, 81± 9 y.o.)<br>10 aMCI patients (6W, 4M, 78 ± 7 y.o.)<br>20 healthy subjects (8W, 12M, 73 ± 5 y.o.)	Decrease of ChP [18F]-FDG accumulation in AD and aMCI patients compared to controls, in relation to disease severity.
**Proteinopathies**			
Lowe et al (2016)	[18F]-AV1451 PET autoradiography in brain samples	35 patients with dementia-related neuropathologies (13W, 22M, 49-91 y.o)	The radiotracer targeted at tau protein aggregates binds to calcifications in ChPs ("off-target" binding)

Ikonomovic et al (2016)	[^{18}F]-AV1451 PET	3 normal controls (1W, 2M, 65-81 y.o)<br>2 Alzheimer's disease patients (77 and 91 y.o.)<br>1 normal control (83y.o.)	Tau protein aggregates found post-mortem in patient ChPs in the same location as radiotracer uptake as demonstrated by the PET images of a distinct patient ("on-target" binding).
Lee et al (2018)	[^{18}F]-FTP PET	23 Black/African American (B/AA) normal controls (19W, 4M, 73.1 ±7.1y.o.)<br>124 White (Wh) normal controls (68W, 56M, 76.5 ±6.8y.o.)	Elevated [^{18}F]-FTP uptake in ChPs of B/AA participants compared to Wh: "off-target" binding to melanin.
Pontecorvo et al (2017)	[^{18}F]-AV-1451 PET	48 AD patients (27W, 21M, 54-95y.o)<br>95 MCI patients (48W, 47M, 50-92y.o.)<br>16 young CNS (7W, 9M, 20-40y.o.)<br>58 older CNS (26W, 32M, 50-87y.o.)	Increase of [^{18}F]-AV-1451 retention in subject ChPs with age, regardless of clinical diagnosis. It could reflect age-related tau aggregations or "off target" binding.
Marquié et al (2017)	[^{18}F]-AV-1451 PET	1 Parkinson disease (PD) patient (M, 71y.o.)	Visualization of high [^{18}F]-AV-1451 retention in patient ChPs with PET, while postmortem analyses show absence of tau aggregates in ChPs; study in favor of the "off-target" hypothesis.
**Immune Response**			
Hirvonen et al (2012)	[^{11}C]-PBR28 PET	16 temporal lobe epilepsy patients (8W, 8M, 36 ± 10y.o.)<br>30 healthy subjects (12W, 18M, 46 ± 14y.o.	Higher TSPO radiotracer uptake in ipsilateral ChPs of epileptic patients compared to contralateral ChPs.

Drake et al (2011)	[^{11}C]-PK11195 PET	4 patients at risk of stroke (1W, 3M, 58-72y.o.)<br>4 healthy subjects (3W, 1M, 58-68y.o.)	TSPO radiotracer uptake in patient ChPs was considered "off-target" and therefore disregarded while at the same time immune cells were shown to be recruited via the ChPs in a rodent model.

[^{11}C]-verapamil: P-glycoprotein-targeted radiotracer; **[^{18}F]-FDG:** glucose metabolism radiotracer; **[^{18}F]-FTP:** tau aggregate-targeted radiotracer**;** **[^{99m}Tc]-sestamibi:** P-glycoprotein-targeted radiotracer**;** **AD:** Alzheimer's disease**;** **ADC:** apparent diffusion coefficient**;** **(a)MCI: (**amnesic) mild cognitive impairment**;** **ASD:** autism spectrum disorder**;** **B/AA:** Black/African American**;** **BOLD fMRI:** blood oxygenation level dependent functional MRI**;** **CE-MRI:** contrast-enhanced MRI**;** **CNS:** cognitive normal subjects**;** **CRPS:** complex regional pain syndrome**;** **CT:** computed tomography**;** **DCE-MRI:** dynamic contrast-enhanced MRI**;** **DSC-MRI:** dynamic susceptibility contrast-enhanced MRI**;** **DTI:** diffusion tensing imaging**;** **DWI:** diffusion weighted imaging**;** **FA:** fractional anisotropy**;** **IIH:** idiopathic intracranial hypertension**;** **M:** man**;** **mCPP:** m-chlorophenylpiperazine 5-HT receptor antagonist**;** **PD:** Parkinson disease**;** **PET:** position emission tomography**;** **QSM:** quantitative susceptibility mapping**;** **SPECT:** single-photon-emission computed tomography**;** **SWI:** susceptibility weighted imaging**;** **T:** Tesla**;** **T1w:** T1-weighted MRI sequence**;** **T2w:** T2-weighted MRI sequence**;** **T2*w:** T2*-weighted MRI sequence**;** **TSPO:** translocator protein 18 kDa**;** **US:** ultrasound**;** **W:** woman**;** **Wh:** White**.**
